# 507. Impact of Baricitinib on Outcomes in Patients Treated with Remdesivir and Dexamethasone for SARS-CoV-2 Pneumonia: A Retrospective Cohort Study

**DOI:** 10.1093/ofid/ofab466.706

**Published:** 2021-12-04

**Authors:** Lauren Dea, Hal Piwonka, Learned Gonzales, Shubha Kerkar, Xolani Mdluli, Yisu Kim

**Affiliations:** Desert Regional Medical Center, California

## Abstract

**Background:**

There is a lack of data specifically addressing the effects of triple therapy consisting of baricitinib plus remdesivir plus dexamethasone compared to dual therapy with remdesivir plus dexamethasone among patients with severe acute respiratory syndrome coronavirus-2 (SARS-CoV-2) pneumonia.

**Methods:**

This retrospective study enrolled hospitalized adults with SARS-CoV-2 receiving supplemental oxygen without invasive mechanical ventilation (IMV) being treated baricitinib (≤10 days) plus remdesivir (≤10 days) plus dexamethasone (≤10 days) or remdesivir (≤10 days) plus dexamethasone (≤10 days). The primary endpoint was 28-day mortality. Secondary objectives of this study were to measure progression to IMV, pulse oximetry (SpO2)/fraction of inspired oxygen (FiO2) from hospitalization to discharge, hospital length of stay (LOS), 14-day mortality, 14-day hospital readmissions, inflammatory markers, and safety outcomes.

**Results:**

Among patients receiving supplemental oxygen without IMV, 28-day mortality for triple therapy vs. dual therapy was 20% and 24%, respectively (P=1.000). The effect of triple therapy compared to dual therapy on lung function was demonstrated by a 76% vs. 25% increase in SpO2/FiO2. This benefit must be contextualized by an increased progression to IMV among patients receiving triple therapy compared to dual therapy (10 patients [50%] vs. 7 patients [28%], respectively; P=0.130). The increased incidence of IMV translated to a significantly longer hospital LOS among patients receiving triple therapy compared to dual therapy (26 days vs. 17 days, respectively; P=0.001).

**Conclusion:**

In patients receiving supplemental oxygen without IMV for SARS-CoV-2, triple therapy was not associated with a clinically meaningful reduction in 28-day mortality when compared to dual therapy.

**Disclosures:**

**All Authors**: No reported disclosures

